# Reinforcement Learning-Based Reactive Obstacle Avoidance Method for Redundant Manipulators

**DOI:** 10.3390/e24020279

**Published:** 2022-02-15

**Authors:** Yue Shen, Qingxuan Jia, Zeyuan Huang, Ruiquan Wang, Junting Fei, Gang Chen

**Affiliations:** School of Modern Post (School of Automation), Beijing University of Posts and Telecommunications, Beijing 100876, China; yuefei@bupt.edu.cn (Y.S.); qingxuan@bupt.edu.cn (Q.J.); zyh214@bupt.edu.cn (Z.H.); wangruiquan@bupt.edu.cn (R.W.); fjt@bupt.edu.cn (J.F.)

**Keywords:** redundant manipulator, obstacle avoidance, reinforcement learning, null space

## Abstract

Redundant manipulators are widely used in fields such as human-robot collaboration due to their good flexibility. To ensure efficiency and safety, the manipulator is required to avoid obstacles while tracking a desired trajectory in many tasks. Conventional methods for obstacle avoidance of redundant manipulators may encounter joint singularity or exceed joint position limits while tracking the desired trajectory. By integrating deep reinforcement learning into the gradient projection method, a reactive obstacle avoidance method for redundant manipulators is proposed. We establish a general DRL framework for obstacle avoidance, and then a reinforcement learning agent is applied to learn motion in the null space of the redundant manipulator Jacobian matrix. The reward function of reinforcement learning is redesigned to handle multiple constraints automatically. Specifically, the manipulability index is introduced into the reward function, and thus the manipulator can maintain high manipulability to avoid joint singularity while executing tasks. To show the effectiveness of the proposed method, the simulation of 4 degrees of planar manipulator freedom is given. Compared with the gradient projection method, the proposed method outperforms in a success rate of obstacles avoidance, average manipulability, and time efficiency.

## 1. Introduction

Compared with traditional robotic manipulators, redundant manipulators have more degrees of freedom (DOF) in joint space than task space, which possesses better flexibility for complicated tasks. Therefore, redundant manipulators are widely used in fields such as human-robot collaboration [1], medical surgery [2], and space exploration [3]. Redundant manipulators often work in dynamic environments and often even share workspaces with people. The manipulator may collide with people or other obstacles during the movement, which requires the capability of real-time obstacle avoidance. In many tasks, such as polishing and welding, the manipulator is obliged to track the desired trajectory under complex physical constraints. As a result, manipulators need to achieve real-time obstacle avoidance while completing given end-effector motion tasks.

Real-time or reactive obstacle avoidance of redundant manipulators has been extensively investigated. However, most studies, such as the artificial potential field method [4,5], mainly focused on obstacle avoidance in point-to-point tasks, where the end-effector motion is not specified. To meet the end-effector trajectory constraint while avoiding obstacles, Jacobian pseudoinverse-based methods [6] have been introduced, in which the gradient projection method (GPM) [7] is the most popularly adopted. The motion of a redundant manipulator is usually divided into end-effector motion and self-motion. The gradient projection method projects the optimization function of the self-motion gradient to the null space of the Jacobian matrix, which can adjust the configuration to avoid obstacles without affecting the end-effector motion. The optimization function usually consists of distance in different forms, such as distance from the key point to obstacles [8] and repulsive potential field generated by obstacles [9]. However, the GPM may lead to joint singularity or joint position limits while avoiding obstacles; thus the manipulator will fail to track the desired trajectory. The damped least-squares method (DLS) [10] and weighted least-norm methods (WLN) [11] are introduced to solve the problem. Combined with DSL and WLN, Zhang [12] presented an improved weighted gradient projection method (IWGPM), in which the manipulator can handle joint singularity and joint position limits, but it increases the tracking error. To coordinate end-effector motion and self-motion, Liu [13] proposed a weighted additional deviation velocity based on the gradient project method (GP-WADV). However, coping with multiple constraints simultaneously is still hard for these methods.

Recently, many studies have applied learning-based methods to obstacle avoidance path planning under multiple constraints. Qureshi [14] proposed a motion planning network using deep learning to generate a collision-free path, but it cannot satisfy real-time obstacle avoidance. Xu [15] formulated the obstacle avoidance problem as quadratic programming (QP), then a deep recurrent neural network is established to solve the QP problem online. Apart from deep learning (DL) methods, deep reinforcement learning (DRL) is considered to be very promising for path planning due to its excellent learning ability. Sangiovanni [16] proposed a real-time collision avoidance approach based on Normalized Advantage Function for safe human-robot coexistence. However, redundancy is not considered. Kumar [17] presented a method that used the Proximal Policy Optimization algorithm to directly map task-space goals into joint-space commands. The method can handle redundancy and joint limits automatically without manually specifying constraints, but the end-effector tracking process is not considered. Hua [18] proposed a method to avoid obstacles for redundant manipulators based on the Deep Deterministic Policy Gradient algorithm, but it is mainly applied in a narrow duct. However, learning-based methods usually neglect the characteristics of redundant manipulators and can only be applied to point-to-point collision-free path planning.

In summary, the existing methods cannot completely satisfy multiple constraints, such as obstacle avoidance and singularity avoidance in trajectory tracking tasks. Motivated by GPM and DRL, we propose a reactive obstacle avoidance method for redundant manipulators. The method leverages the null space of GPM and the learning ability of DRL, which is very suitable for obstacle avoidance of redundant manipulators under multiple constraints. The major contributions of this paper are as follows:

(1) A general DRL framework for obstacle avoidance of redundant manipulators is established, in which multiple constraints can be integrated easily.
(2) An improved representation of the state is given in obstacle avoidance. The dimension of state space is independent of the distribution of obstacles. Therefore, the learned obstacle avoidance strategy has a good generalization.
(3) The self-motion of redundant manipulators is utilized to reduce the action space from the entire joint space to the null space of the Jacobian matrix, which greatly improves the learning efficiency of DRL.
(4) A novel reward function of reinforcement learning is designed to cover multiple constraints. The manipulability of a manipulator is introduced, so the manipulator can learn to avoid obstacles while keeping away from the joint singularity.

The rest of this paper is organized as follows: Section 2 defines the obstacle avoidance problem of redundant manipulators. Section 3 describes our proposed reactive obstacle avoidance method. Section 4 presents the experiments and results. Section 5 summarizes the research work.

## 2. Problem Setup

This paper focuses on the situation in which a redundant manipulator works in a populated industrial environment, possibly invaded by obstacles. A collision could occur while the robot is reaching its target or executing a specific task. The manipulator aims to avoid surrounding obstacles while tracking the desired trajectory.

**Figure 1:**
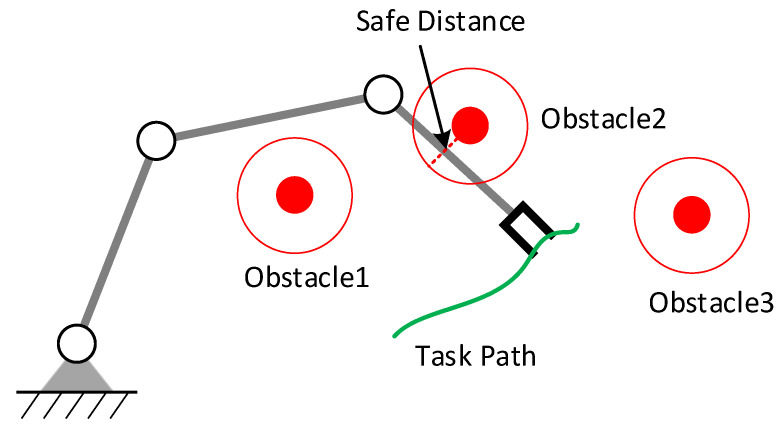
Obstacle avoidance for a redundant manipulator.

We assume that obstacles will not appear on the desired trajectory of the manipulator; otherwise, the manipulator needs to stop or re-specify the desired motion of the end-effector. Multiple obstacles may appear in the workspace; the manipulator only needs to react when the distance between the manipulator and the obstacle is less than a specified safety distance, which can be called reactive obstacle avoidance. As shown in Figure 1, the manipulator only needs to avoid Obstacle 2 when tracking the desired trajectory.

When the dimension *n* in the joint space of the manipulator is greater than the dimension *m* in task space, the manipulator has *r* = *n* − *m* degrees of redundancy for the task. The relationship between the movement of the joint space and the task space is given in Equation (1):

(1) $$\dot{x}=J\dot{q}$$

where $\dot{x}\in R^{m}$ is the end-effector velocity in task space, $\dot{q}\in R^{n}$ is the joint velocity in joint space, and $J\in R^{m\times n}$ is the Jacobian matrix.

The inverse solution of redundant manipulators is not unique, which means that infinite joint configurations can reach the same end-effector pose. According to the GPM, the inverse solution can be expressed as Equation (2):

(2) $$\dot{q}=J^{†}\dot{x}+(I-J^{†}J)\dot{\varphi }$$

where $J^{†}\in R^{n\times m}$ is the Moore–Penrose pseudo-inverse, $I-J^{†}J\in R^{n\times n}$ represents the null space of the Jacobian matrix, and $\dot{\varphi }\in R^{n}$ is an arbitrary joint velocity vector. The first item is the minimum norm solution, which is used to track the end-effector trajectory. The second item is the homogeneous general solution. It refers to self-motion, which can meet other requirements, such as obstacle avoidance. Obviously, the first item can be easily obtained based on the desired end-effector motion. This paper mainly studies the optimization for the second item.

## 3. Method

According to Equation (2), the gradient projection method realizes obstacle avoidance through defining $\dot{\varphi }$ in various forms. The manipulator may be singular or exceed joint position limits while avoiding obstacles. We propose a DRL-based method to learn $\dot{\varphi }$ by a neural network, which can handle multiple constraints automatically.

### 3.1. Reinforcement Learning

**Figure 2:**
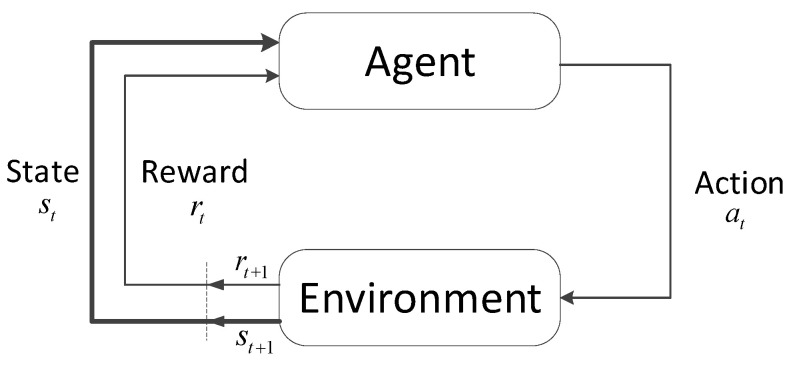
Reinforcement learning framework.

Reinforcement learning (RL) refers to the idea that the agent optimizes its action through interaction with the environment. The RL framework is shown in Figure 2.

At each time step *t*, the agent is in the state $s_{t}\in S$, then takes an action $a_{t}\in A$ according to a policy π(a|s) mapping from state to action. Each action affects the environment and hence changes its state to $s_{t+1}$. The agent receives a reward $r_{t+1}\in R$ at time step *t*+1. The goal of the agent is to find the optimal policy $\pi^{*}$ that maximizes the total expected reward $G_{t}$ with a discount factor γ. The expression of $G_{t}$ and $\pi^{*}$ are given in Equations (3) and (4), respectively.

(3) $$G_{t}=r_{t+1}+\gamma r_{t+2}+\gamma^{2}r_{t+3}+\ldots =\sum_{k=0}^{\infty }\gamma^{k}r_{t+k+1}$$

(4) $$\pi^{*}=\arg\;\underset{\pi }{\max}E\left[\sum_{k=0}^{\infty }\gamma^{k}r_{t+k+1}\right]$$

To find the optimal policy, there are mainly three types of methods: valued-based, policy-based, and actor-critic methods. In value-based methods, the optimal value function is first estimated, and then the optimal policy is derived from the value function. A typical method is Deep Q-Network (DQN) [19], which uses a neural network to estimate the optimal value function. In policy-based methods, the optimal policy is estimated directly from the experiences of the agent. A typical method is REINFORCE [20], which uses the policy gradient to find the optimal policy. The actor-critic methods combine the characteristics of two types of methods, using the actor for policy estimation and the critic for value function estimation. Typical methods are the Deep Deterministic Policy Gradient (DDPG) [21], Trust Region Policy Optimization (TRPO) [22], Proximal Policy Optimization (PPO) [23], Asynchronous Advantage Actor-Critic (A3C) [24], and Soft Actor-Critic (SAC) [25]. In the field of robotics, the state space and action space are generally continuous, and actor-critic methods are widely used.

For reinforcement learning problems, the design of state, action, and reward functions are very important, which directly affect the training effect.

### 3.2. State Definition

For obstacle avoidance of the manipulator, the state should include the position information of the manipulator and obstacles. Intuitive design for the state is $s=\{q,x_{o}\}$, where $q\in R^{n}$ is the joint angles of the manipulator and $x_{o}\in R^{m}$ is the position of the obstacle. The definition of the state remains as a disadvantage because it needs to expand the dimension when multiple obstacles exist. Therefore, the dimension of state space will be relevant to the number of obstacles, leading the neural network trained to have no generalization.

To solve the problem, we convert the position of obstacles to the distance vector from each link to its closest obstacle. Specifically, let O be the set of points on the surface of all obstacles, and let ${ℒ}_{i}$ be the set of points of the ith link. Let $o_{i}\in O$ and $l_{i}\in {ℒ}_{i}$ be the closest points in the two sets, as given in Equation (5).

(5) $$\left\|o_{i}-l_{i}\right\|=\underset{o\in O}{\min}\underset{l\in {ℒ}_{i}}{\min}\left\|o-l\right\|$$

**Figure 3:**
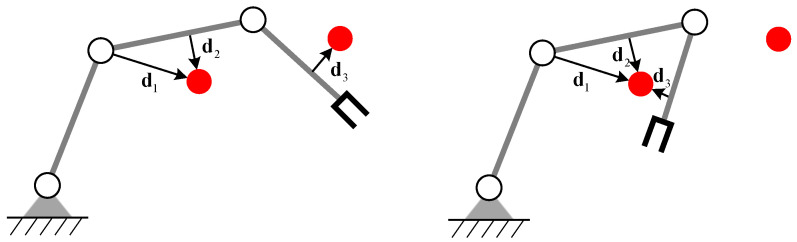
The closet distance vector for each link.

The vector for each link connecting these closest points is defined as $d_{i}=o_{i}-l_{i}$. The dimension of $d_{i}$ is 2 for planar manipulators or 3 for spatial manipulators. The closest distance vector for a 3-DOF planar manipulator is shown in Figure 3.

Based on the closest distance vector, we redefine the state of the obstacle avoidance as follows.

(6) $$s=\{q,d_{1},d_{2},\ldots ,d_{n}\}$$

The state definition realizes the general position representation of multiple obstacles. When the number or distribution of obstacles in the environment changes, the state dimension remains unchanged. In addition, because the distance between each link and its closest obstacle is recorded, it is helpful for the neural network to learn the coordination for obstacle avoidance among the links of the manipulator.

### 3.3. Action Definition

The action definition cannot directly adopt the joint velocity of the configuration space, which fails to satisfy the self-motion constraint. According to Equation (2), the action is naturally defined in Equation (7).

(7) $$a=\{\dot{\varphi }\}$$

Combing the action with the null space of the Jacobian matrix, the manipulator can avoid obstacles by adjusting the joint angle while keeping the end-effector position unchanged.

### 3.4. Reward Function Design

The design for the reward function is indeed important for reinforcement learning, and the reward function should guide the manipulator to learn the optimal strategy. In addition to avoiding obstacles, the manipulator should also consider other constraints, such as joint singularity avoidance and joint position limits. The reward function contains three items.

The obstacle avoidance item is defined as Equation (8). When the closest distance between the manipulator and obstacles is less than the safe distance $d_{s}$, a negative reward will be generated.

(8) $$r_{o}=\sum_{i=1}^{n}\min(\left\|d_{i}\right\|/d_{s}-1,0)$$

The joint motion item is defined as Equation (9). The joint position increment of the manipulator is required to be as small as possible.

(9) $$r_{a}=-\left\|(I-J^{†}J)a\right\|$$

The joint singularity item is defined as Equation (10). Manipulability [26], introduced by Yoshikawa, is a common index to measure singularity. A higher manipulability is good for tracking variable trajectories.

(10) $$r_{m}=\sqrt{\det(JJ^{T})}$$

In summary, the reward function is designed as Equation (11).

(11) $$r=\lambda_{1}r_{o}+\lambda_{2}r_{a}+\lambda_{3}r_{m}$$

where $\lambda_{1},\lambda_{2},\lambda_{3}$ represent the weight of each item, respectively. The corresponding values are given in Section 4. It should be noticed that if a collision occurs or any joint exceeds its position limits, then a negative reward r=−10 will be generated.

### 3.5. Learning for Reactive Obstacles Avoidance

**Figure 4:**
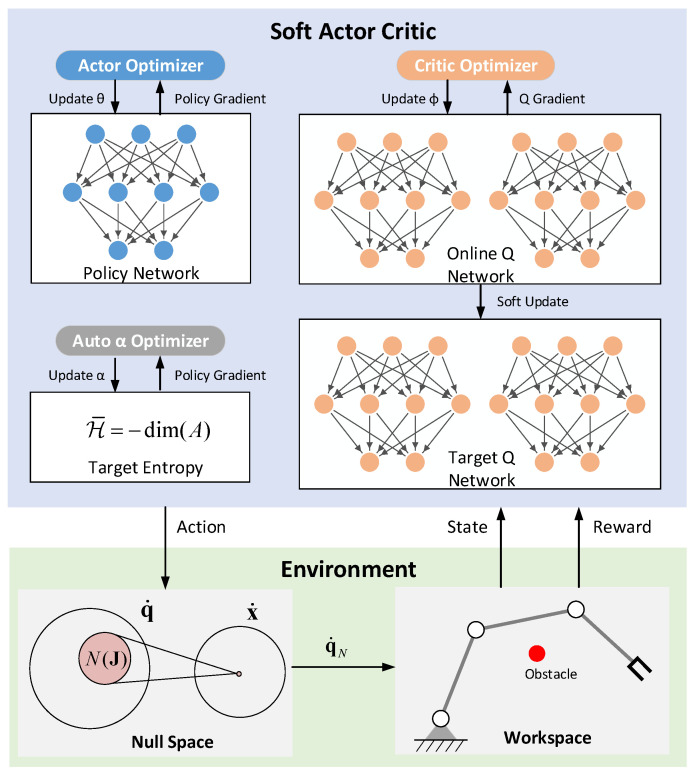
Framework of reactive obstacle avoidance.

Similar to the RL framework in Section 3.1, the reactive obstacle avoidance framework is divided into two parts: agent and environment. The difference lies in where the environment part has a null space module for reactive obstacle avoidance, as shown in Figure 4.

#### 3.5.1. SAC Algorithm

To avoid obstacles and maintain high manipulability during the movement, sufficient exploration in the state space is encouraged under multiple constraints. SAC, one of the state-of-the-art deep reinforcement learning algorithms, is widely used for its good exploration. Therefore, SAC is very suitable for obstacle avoidance of redundant manipulators.

Instead of maximizing the discounted cumulative reward, SAC introduces the entropy of the policy, as shown in Equation (12).

(12) $$\pi^{*}=\arg\;\underset{\pi_{\theta }}{\max}E\left[\sum_{t}\gamma^{t}\left(r(s_{t},a_{t})+\alpha ℋ\left(\pi_{\theta }(\cdot |s_{t})\right)\right)\right]$$

where θ represents parameters of the policy, α is the temperature parameter for regulating the entropy term against the reward, and ℋ denotes the entropy of the policy.

In SAC, the Q function $Q_{\phi }(s,a)$ and policy $\pi_{\theta }$ are approximated by deep neural networks, which can be learned with the stochastic gradient descent method. The *Q* function can be learned by minimizing the soft Bellman residual, as shown in Equation (13).

(13) $$J_{Q}(\phi )=E\left[{\left(Q_{\phi }(s_{t},a_{t})-r(s_{t},a_{t})-\gamma E_{s_{t+1}}[V_{\tilde{\phi }}(s_{t+1})]\right)}^{2}\right]$$

where $V_{\tilde{\phi }}(s)=E_{\pi_{\theta }}[Q_{\tilde{\phi }}(s,a)-\alpha \log\pi_{\theta }(a|s)]$, and $Q_{\tilde{\phi }}$ is a target *Q* network, whose parameter $\tilde{\phi }$ is obtained as an exponentially moving average of ϕ. Moreover, the policy $\pi_{\theta }$ can be learned by minimizing the expected KL-divergence, as shown in Equation (14).

(14) $$J_{\pi }(\theta )=E_{s\sim D}\left[E_{a\sim \pi_{\theta }}[\alpha \log\pi_{\theta }(a|s)-Q_{\phi }(s,a)]\right]$$

where D is the replay buffer for storing experiences of the agent.

Finally, SAC also provides an automatic way to update the temperature parameter α, as shown in Equation (15).

(15) $$J(\alpha )=E_{a\sim \pi_{\theta }}[-\alpha \log\pi_{\theta }(a|s)-\alpha \bar{ℋ}]$$

where $\bar{ℋ}$ is a hyperparameter interpreted as the target entropy. In continuous action tasks, such as most robotic tasks, $\bar{ℋ}$ is usually defined as the negative of the action dimension.

The SAC algorithm is summarized in Algorithm 1.
**Algorithm 1**. Soft Actor-Critic (SAC)1. Initialize policy network θ, Q network $\phi_{1},\phi_{2}$, target Q network ${\tilde{\phi }}_{1}=\phi_{1},{\tilde{\phi }}_{2}=\phi_{2}$ 2. Initialize replay buffer D=∅ 3. **for** each epoch **do** 4.     **for** each environment step **do** 5.       Sample $a_{t}$ from $\pi_{\theta }(\cdot |s)$, collect $r_{t},s_{t+1}$ 6.       $D=D\cup \{s_{t},a_{t},r_{t},s_{t+1}\}$ 7.     **end for** 8.     **for** each gradient step **do** 9.        $\phi_{i}=\phi_{i}-\lambda_{Q}\nabla J_{Q}(\phi_{i})$, for i∈{1,2} 10.       $\theta =\theta -\lambda_{\pi }\nabla J_{\pi }(\theta )$ 11.       $\alpha =\alpha -\lambda_{\alpha }\nabla J(\alpha )$ 12.       ${\tilde{\phi }}_{i}=(1-\tau )\phi_{i}+\tau {\tilde{\phi }}_{i}$, for i∈{1,2} 13.     **end for** 14. **end for**

#### 3.5.2. RL-Based Reactive Obstacle Avoidance Algorithm for Redundant Manipulators

The redundant manipulator senses the position of obstacles in real-time and moves in the environment. Then the reward is calculated according to Equation (11) and is transmitted to the SAC agent. The actor and critic networks update the parameters from the experiences of the agent. The final action $\dot{\varphi }$ is output through the actor network. Combined with the null space of the Jacobian matrix, the joint velocity of the self-motion can be obtained as Equation (16).

(16) $${\dot{q}}_{N}=(I-J^{†}J)\dot{\varphi }$$

According to the expected motion ${\dot{x}}_{D}$ of the manipulator end-effector, the minimum norm solution is obtained as Equation (17).

(17) $${\dot{q}}_{D}=J^{†}{\dot{x}}_{D}$$

Finally, the joint velocity of the manipulator can be expressed as Equation (18):

(18) $$\dot{q}={\dot{q}}_{D}+{\dot{q}}_{N}$$

The joint angle updates through time integration, and then a new state can be generated. The process is repeated until the task of the manipulator is finished.

The RL-based reactive obstacle avoidance algorithm for redundant manipulators is summarized in Algorithm 2.
**Algorithm 2**. Proposed Obstacle Avoidance Algorithm for Redundant Manipulators1. Obtain state $s=\{q,d_{1},d_{2},\ldots ,d_{n}\}$ 2. Calculate the minimum distance $d_{\min}={\left\{\left\|d_{1}\right\|,\left\|d_{2}\right\|,\ldots ,\left\|d_{n}\right\|\right\}}_{\min}$ 3. **while** $d_{\min}<d_{s}$, **do** 4.     $\dot{\varphi }=SAC.Actor(s)$ 5.     $\dot{q}=J^{†}{\dot{x}}_{D}+(I-J^{†}J)\dot{\varphi }$ 6.        **if** $\dot{q}$ is out of joint velocity range, **then** 7.           $\dot{q}=scaled\;\dot{q}$
8.        **end if** 9.      $q=q+\dot{q}\cdot \Delta t$ 10.     $s=\{q,d_{1},d_{2},\ldots ,d_{n}\}$ 11.     $d_{\min}={\left\{\left\|d_{1}\right\|,\left\|d_{2}\right\|,\ldots ,\left\|d_{n}\right\|\right\}}_{\min}$ 12. **end while**

#### 3.5.3. Training Strategy

**Figure 5:**
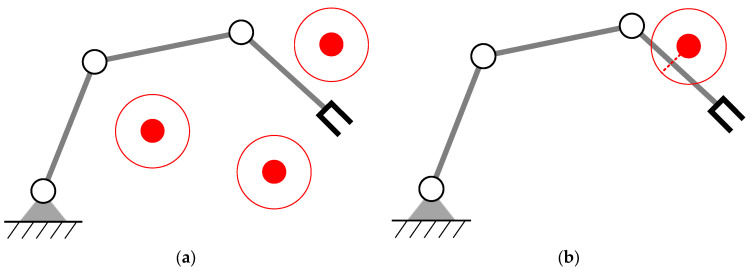
Methods to generate obstacles: (**a**) Obstacles randomly generated in the workspace; (**b**) An obstacle generated in the safe distance.

An intuitive training strategy is to randomly generate multiple obstacles in the environment and let the manipulator interact with the environment to learn to avoid obstacles, as shown in Figure 5a. However, this method will generate many cases where obstacles are far from the safe distance. Thus, the manipulator does not need to respond, leading to useless learning. To improve the learning efficiency of obstacle avoidance, obstacles can be generated directly near the links of the manipulator; that is, in the initial state, obstacles have invaded the safe distance of the manipulator. In addition, considering that in most cases there is only one obstacle in the safety distance of the manipulator at a moment, only one obstacle is generated during training, as shown in Figure 5b.

When the end-effector of the manipulator is fixed, there are infinite distributions of obstacles. Except for learning obstacle avoidance in this situation, the RL agent should adapt to changes in the end-effector position. Learning varying obstacle distribution and end-effector positions simultaneously is very difficult. Therefore, we design a two-stage learning strategy motivated by curriculum learning [27]. In Stage I, the manipulator starts from a fixed configuration and learns to avoid obstacles in null space. In Stage II, at the beginning of each training, the end-effector of the manipulator will move to a new random position nearby. After the training of Stage I is completed, its network parameters are used to initialize the network of Stage II.

## 4. Results and Discussion

To evaluate the performance of our method and the gradient projection method, two scenarios have been carried out. All simulations were run on a computer with a 3.5 GHz Intel(R) Xeon(R) E5-1620 v3 processor and 16 GB RAM.

### 4.1. System Description

The simulation environment is built on OpenAI gym [28]. It contains a 4-DOF planar redundant manipulator and random obstacles. Each link length of the manipulator is 1 m. The ranges of joint position and joint velocity are listed in Table 1.

**Figure 6:**
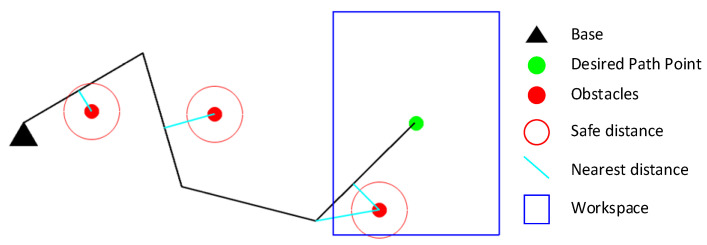
The 4-DOF planar redundant manipulator.

The manipulator executes tasks in the blue workspace (1.2 m × 1.6 m). The task only constraints the end-effector position, so there are two redundant joints. When the manipulator is tracking the desired trajectory, some obstacles in the environment may invade the safe distance (0.2 m) of the manipulator. The shape of the obstacle can vary. Considering that the distance between the manipulator link and its closest obstacle has been calculated, the obstacle can be simplified as a circle. The 4-DOF planar manipulator is shown in Figure 6.

### 4.2. Parameters Selection

**Figure 7:**
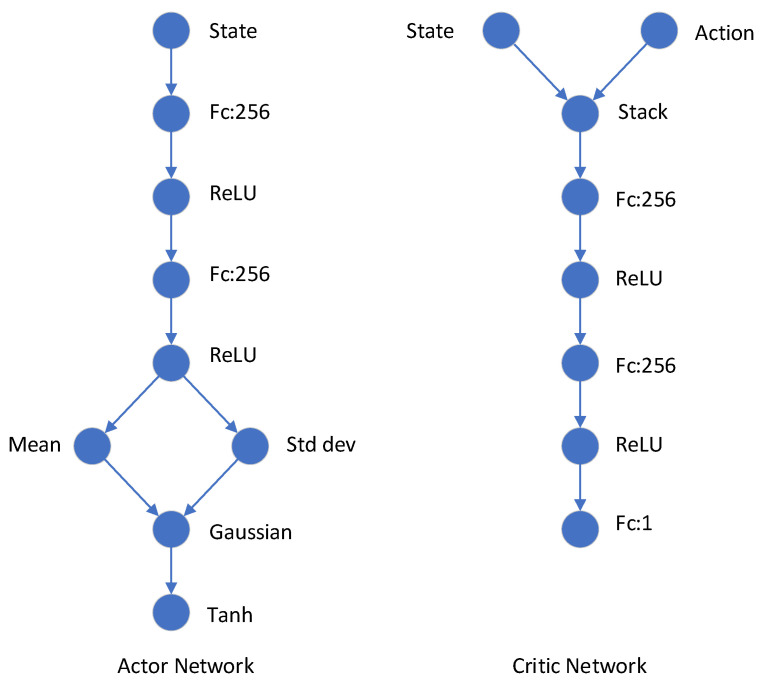
Network structure of the SAC.

The training of the SAC algorithm mainly has two types of parameters: network structure parameters and training process parameters. The specific network of the SAC algorithm is shown in Figure 7. The entire network is composed of fully connected layers, and ReLU is used for the activation function.

The main parameters of the training process are shown in Table 2. The training process only uses the CPU.

### 4.3. Training

**Figure 8:**
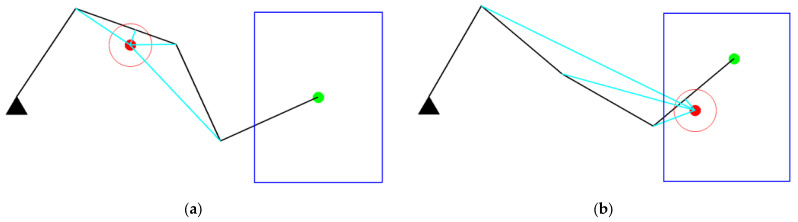
(**a**) Training in Stage I; (**b**) Training in Stage II.

According to the training strategy in Section 3.5.3, the training is divided into two stages, as shown in Figure 8. In Stage I, the position of the end-effector is fixed. In Stage II, at the beginning of each training episode, the end-effector of the manipulator will perform a wandering with a radius of R = 0.1 m.

**Figure 9:**
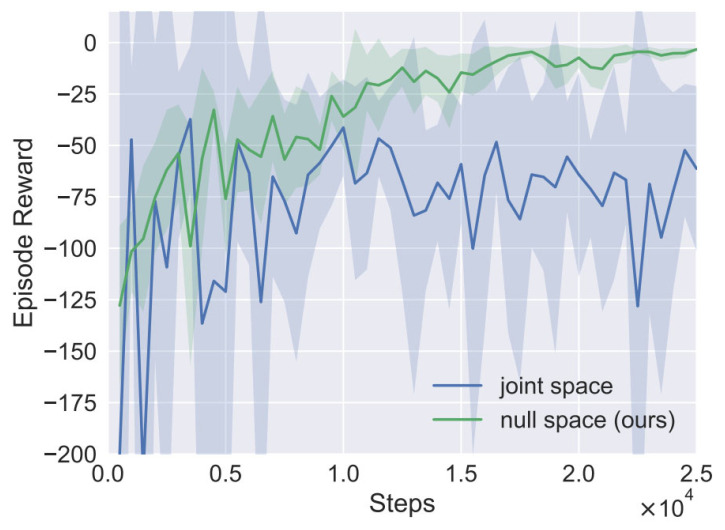
Learning curve of episode reward.

At the beginning of each training episode, an obstacle is generated around the link of the manipulator. The manipulator continuously learns to avoid obstacles during the interaction with the environment until the closest distance between the manipulator and the obstacle is out of the safe distance or the interaction number exceeds 400. A total of 50 epochs are trained, and each epoch contains 500 environment interactions. The green curve in Figure 9 shows the total average return of evaluation during training for SAC. We use five different random seeds, with each averaging 10 evaluation episodes after every epoch. The solid curve corresponds to the mean and the shaded region to the minimum and maximum returns over the five trials. After 20,000 steps of environment interaction (~40 min), it converges and has a good performance.

We also trained a SAC agent that the action is directly defined as joint velocity in joint space. Because there is no null space constraint, the end-effector of the manipulator cannot remain unchanged while avoiding obstacles. As shown in Figure 9, the training curve in blue cannot even converge, which implies the importance of the action definition.

### 4.4. Simulation and Discussion

To validate our method, two different scenarios have been carried out in simulation with the same 4-DOF planar manipulator. We compared the performance of our method in these scenarios to the GPM [8].

Scenario I: A single obstacle invades the safe distance of the manipulator. This is the most common scenario in which a worker usually approaches the manipulator.

Scenario II: Two obstacles invade the safe distance of the manipulator simultaneously. This scenario is more challenging, and the manipulator may be too constrained to avoid obstacles.

It should be noticed that many obstacles may appear in the workspace, but the manipulator only needs to react to the obstacles that invade the safe distance.

- Case study in Scenario I

The experiments of Cases A and B are aimed at verifying the obstacle avoidance capability when a single obstacle invades the safe distance of the manipulator.

(1) Case A: The manipulator is required to keep the end-effector stationary.

**Figure 10:**
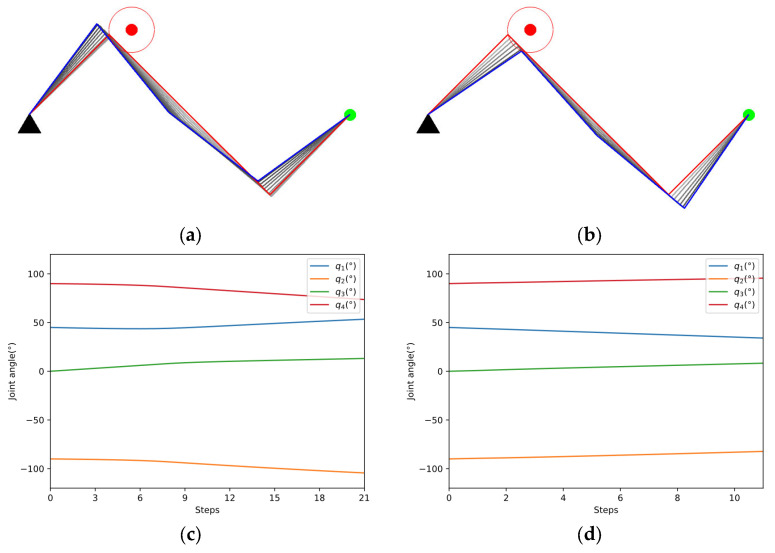

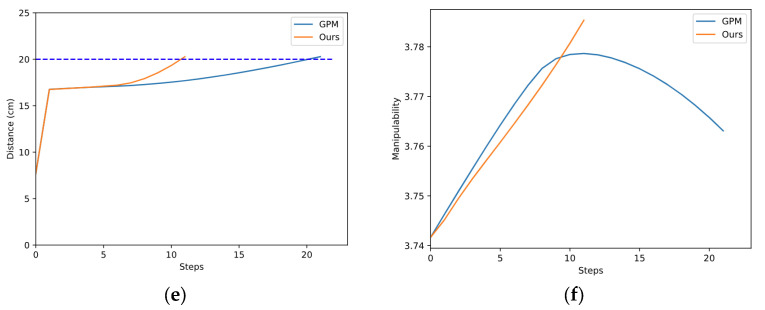
Case A study. (**a**) Obstacle avoidance of GPM; (**b**) Obstacle avoidance of our method; (**c**) Joint angle changes of GPM; (**d**) Joint angle changes of our method; (**e**) Comparison of closest distance to obstacle; (**f**) Comparison of manipulability.

As shown in Figure 10a,b, the two methods start from the same initial configuration $q_{ini}={\left[45{}^{\circ},-90{}^{\circ},0{}^{\circ},90{}^{\circ}\right]}^{T}$ and eventually succeed in avoiding obstacles. The red lines indicate the initial configuration, while the blue lines represent the final configuration. The color of the links changed from light to dark shows the whole process of avoiding obstacles. The blue dotted line in Figure 10e represents the safe distance. According to Figure 10e,f, our method avoids the obstacle in fewer steps and ends with a higher manipulability than GPM.

**Figure 11:**
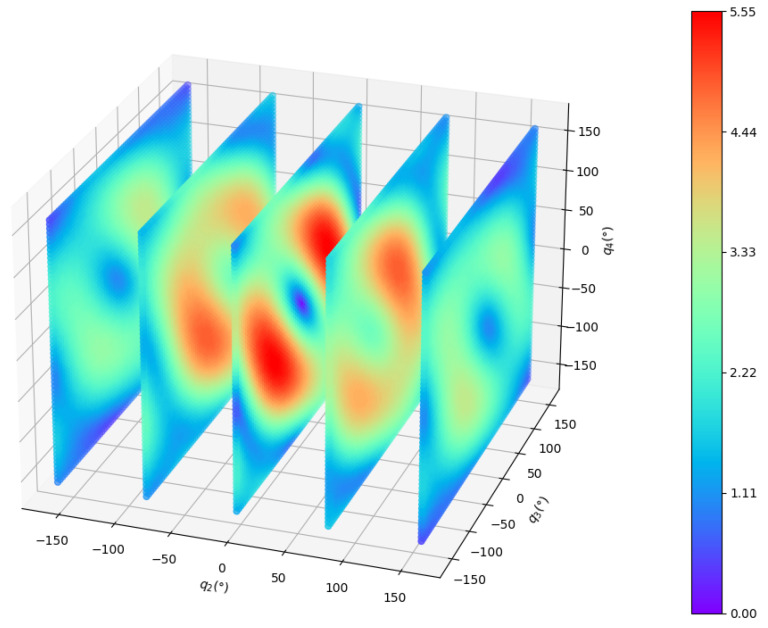
Manipulability of the 4-DOF planar manipulator.

**Figure 12:**
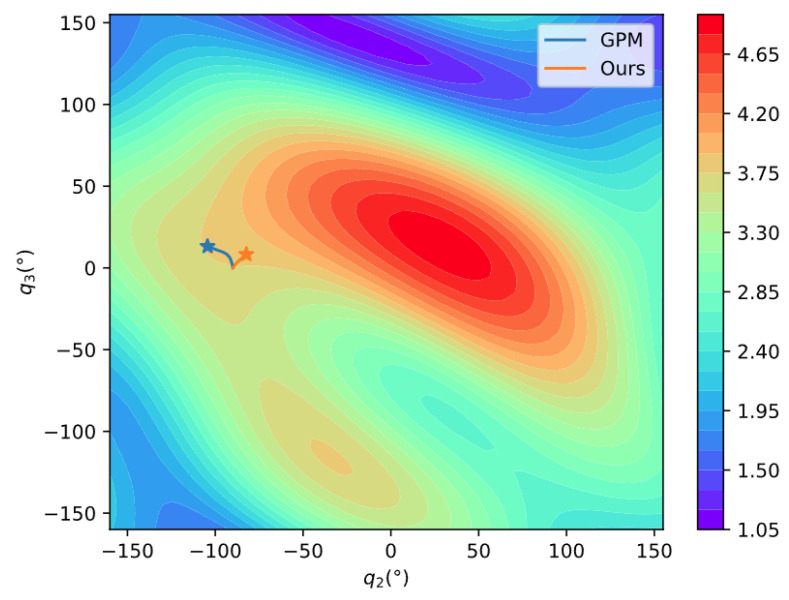
Comparison of manipulability movement.

The manipulability of the 4-DOF planar manipulator is shown in Figure 11. Because the manipulability of a planar manipulator is independent of joint 1, we use $q_{2}\sim q_{4}$ to draw the figure. To show it more clearly, $q_{2}$ is sliced every 80° while $q_{3}$ and $q_{4}$ are sliced every 5°. The distribution of manipulability is relatively complicated. To further compare the performance of the two methods, the manipulability during the process is projected to the plane $q_{2}\times q_{3}$, where $q_{1}=45{}^{\circ},q_{4}=90{}^{\circ}$ at the initial position. Figure 12 clearly shows that our method can move in a direction with higher manipulability, which further demonstrates the search capability in a complex space of our method.

(2) Case B: The manipulator is required to track a line.

**Figure 13:**
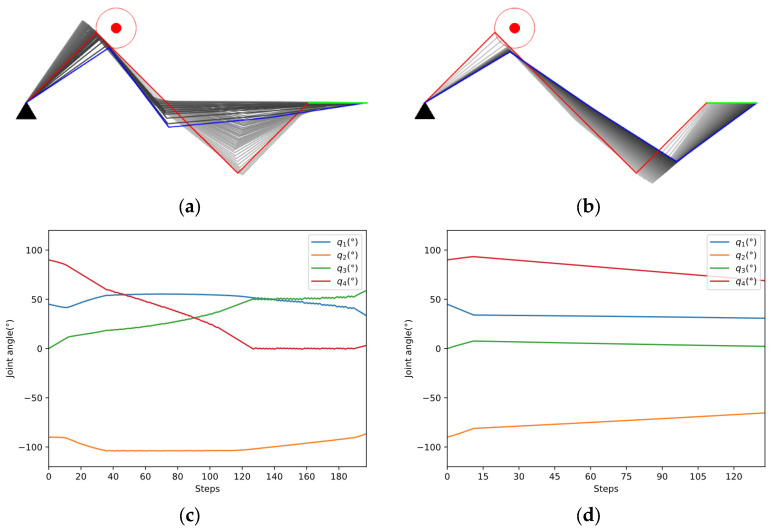

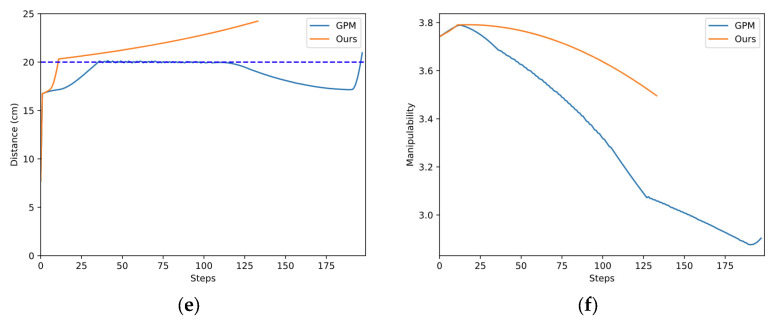
Case B study. (**a**) Obstacle avoidance of GPM; (**b**) Obstacle avoidance of our method; (**c**) Joint angle changes of GPM; (**d**) Joint angle changes of our method; (**e**) Comparison of closest distance to obstacle; (**f**) Comparison of manipulability.

As shown in Figure 13a,b, the two methods start from the same initial configuration $q_{ini}={\left[45{}^{\circ},-90{}^{\circ},0{}^{\circ},90{}^{\circ}\right]}^{T}$ and eventually succeed in tracking the line in green color. Figure 13c,d indicates that the joint changes more smoothly in our method. In Figure 13e, the manipulator applied in our method avoids the obstacle quickly, while the GPM struggles in avoiding the obstacle and tracking the line simultaneously. According to Figure 13f, the manipulability of the two methods both decreased due to more constraints in the tracking task, but the decline of our method is smaller.

- Case study in Scenario II

The experiments of Cases C and D are aimed at verifying the obstacle avoidance capability when two obstacles invade the safe distance of the manipulator.

(1) Case C: The manipulator is required to keep the end-effector stationary.

**Figure 14:**
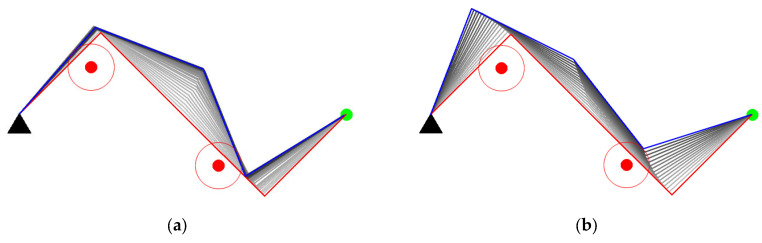

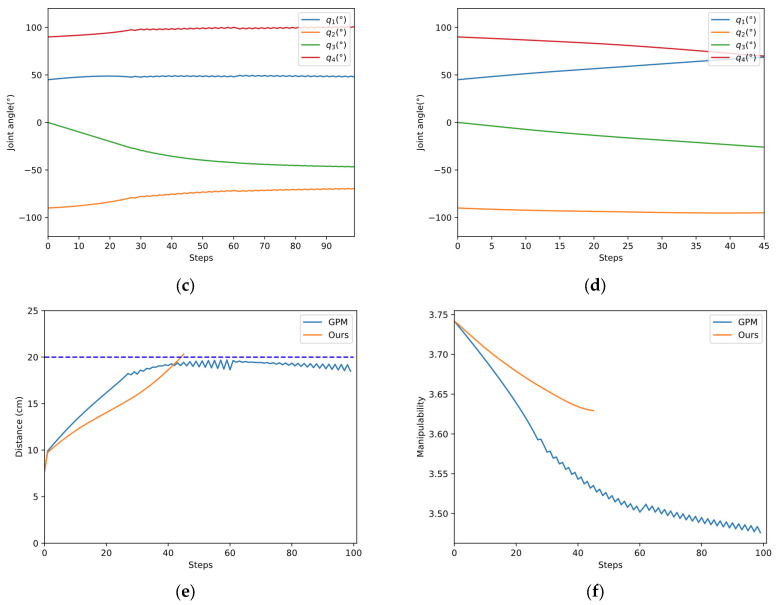
Case C study. (**a**) Obstacle avoidance of GPM; (**b**) Obstacle avoidance of our method; (**c**) Joint angle changes of GPM; (**d**) Joint angle changes of our method; (**e**) Comparison of closest distance to obstacle; (**f**) Comparison of manipulability.

As shown in Figure 14a,b, the two methods start from the same initial configuration. Our method succeeds in avoiding obstacles while the GPM fails. According to Figure 14c,e, the manipulator applied in the GPM oscillates while avoiding obstacles. The reason for the oscillation is that the GPM only considers the influence of the closest invading obstacle. When the manipulator encounters two obstacles, the closest invading obstacle may constantly switch from one to another. The manipulator can be stuck in a dilemma due to the adverse effects of the two obstacles, leading to obstacle avoidance failure. Our method utilizes the distance between each link and its closest obstacle, which ordinates the movement of each joint in null space, then the manipulator successfully avoids the obstacles. In Figure 14f, the manipulability of the two methods both decreased due to more constraints in obstacle avoidance, but the decline of our method is smaller than the GPM.

(2) Case D: The manipulator is required to track a line.

**Figure 15:**
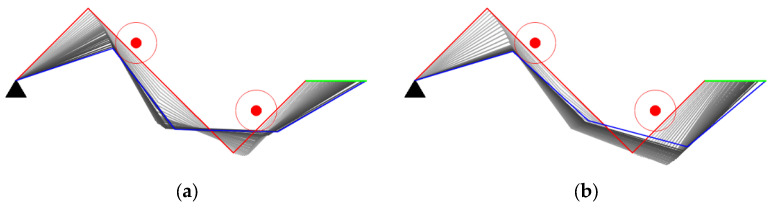

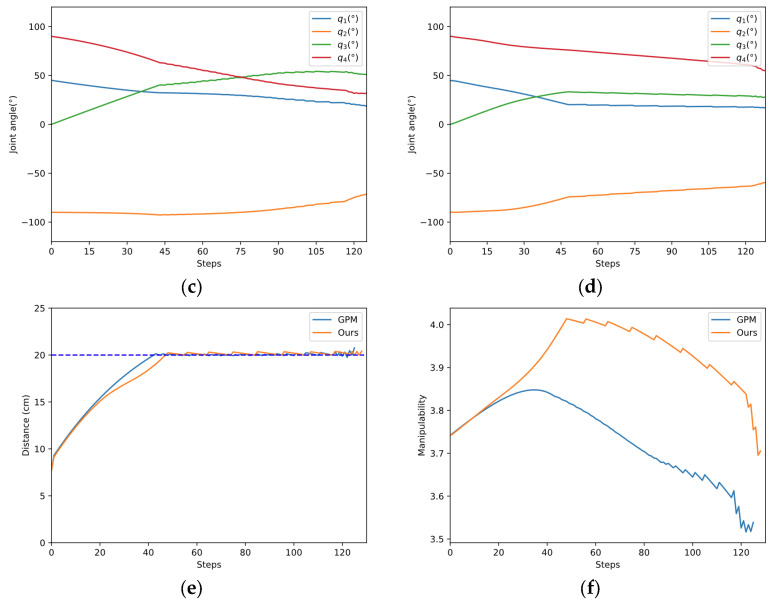
Case D study. (**a**) Obstacle avoidance of GPM; (**b**) Obstacle avoidance of our method; (**c**) Joint angle changes of GPM; (**d**) Joint angle changes of our method; (**e**) Comparison of closest distance to obstacle; (**f**) Comparison of manipulability.

Case D is the most challenging in all cases. The manipulator is required to track a line when two obstacles invade the safe distance. As shown in Figure 15, although the two methods all succeed in tracking the line in green color, joint oscillation exists in the process. It should be noticed that our method has less oscillation (Figure 15c,d) and maintains a higher manipulability (Figure 15f) than the GPM.

- More comparisons

Except for the case study, we provide more general comparisons about the success rate, average manipulability, and time efficiency. Considering that our method differs mainly in null space motion from the GPM, we only evaluate the cases that the end-effector keeps still.

According to the obstacle generation method in Section 4.3, a single obstacle and two obstacles are randomly generated 1000 times near the manipulator links in the initial configuration $q_{ini}={\left[45{}^{\circ},-90{}^{\circ},0{}^{\circ},90{}^{\circ}\right]}^{T}$. The success rate of obstacle avoidance, the average manipulability $\bar{m}$ under successful obstacle avoidance and the time to calculate ${\dot{q}}_{N}$ in two scenarios are compared.

As shown in Table 3, the success rate of the two methods can both reach 100% in Scenario I, but our method has a higher average manipulability while avoiding obstacles. In Scenario II, the success rate of our method is 96.8%, which is nearly 20% higher than the GPM, and our method still achieves a higher average manipulability. One reason for the failure of obstacle avoidance is that the manipulator cannot avoid obstacles only in the null space due to over-constraint, which can be seen in Case C above. In addition, in the harder, Scenario II, the average manipulability of the two methods decreased. The result can be interpreted that more obstacles that invade the safe distance will demand more requirements for obstacles avoidance, leading to less optimization for manipulability. As for time efficiency, our method calculates null space motion faster because it only needs a forward propagation of neural network in a reaction for obstacle avoidance. Moreover, our method saves ~22% more time in Scenario I and ~33% more time in Scenario II than GPM, which indicates that the calculation time of our method grows slower than the GPM when the scenario becomes more complex.

## 5. Conclusions

In this paper, we propose a reactive obstacle avoidance method for redundant manipulators based on DRL. Except for obstacle avoidance, the proposed method can handle joint singularity and joint position limits automatically while tracking the desired task trajectory. We establish a general DRL framework for obstacle avoidance of redundant manipulators, in which a null space module is introduced, and the SAC algorithm is used to train. An improved state definition is used to represent multiple obstacles. The motion in null space is defined as the action. A novel reward function is designed to meet multiple constraints. The simulation results show the effectiveness of our method. Compared with the gradient projection method, our method outperforms in the success rate of obstacle avoidance, average manipulability, and time efficiency. When two obstacles invade the safe distance of the manipulator simultaneously, our method achieves a 96.8% success rate of obstacles avoidance, which is nearly 20% higher than the gradient projection method.

Further research can be conducted based on this paper. The joint speed and obstacles speed can be considered so that the manipulator can avoid obstacles in advance. Except for the speed level, dynamic constraints of motion can also be considered.

## Figures and Tables

**Table 1:** Joint range of the manipulator.

Joint Range	$q_{\min}\;({}^{\circ})$	$q_{\max}\;({}^{\circ})$	${\dot{q}}_{\min}\;({}^{\circ}/s)$	${\dot{q}}_{\max}\;({}^{\circ}/s)$
joint 1	−120	120	−20	20
joint 2	−160	160	−20	20
joint 3	−160	160	−20	20
joint 4	−160	160	−20	20

**Table 2:** Parameters of training.

Parameter	Value
Optimizer	Adam
Learning rate	0.001
Discount factor	0.99
Polyak update factor	0.995
Entropy target	−4
Replay buffer size	1 × 10^5^
Mini-batch size	100
Max episode length	400
$\lambda_{1}$	1
$\lambda_{2}$	0.2
$\lambda_{3}$	0.05

**Table 3:** Comparison of the GPM and our method.

Comparison	GPM	Ours
Success rate in Scenario I	100%	100%
Success rate in Scenario II	77.4%	96.8%
$\bar{m}$ in Scenario I	3.78	3.95
$\bar{m}$ in Scenario II	3.63	3.72
Time to calculate ${\dot{q}}_{N}$ in Scenario I	1.484 ms	1.155 ms
Time to calculate ${\dot{q}}_{N}$ in Scenario II	2.048 ms	1.372 ms

## Data Availability

Not applicable.
